# Minimally destructive hDNA extraction method for retrospective genetics of pinned historical Lepidoptera specimens

**DOI:** 10.1038/s41598-024-63587-7

**Published:** 2024-06-05

**Authors:** Enrique Rayo, Gabriel F. Ulrich, Niklaus Zemp, Michael Greeff, Verena J. Schuenemann, Alex Widmer, Martin C. Fischer

**Affiliations:** 1 Institute of Integrative Biology (IBZ), ETH Zurich, Zurich, Switzerland; 2 Institut Für Veterinärpathologie, University of Zurich, Zurich, Switzerland; 3 Genetic Diversity Centre (GDC), ETH Zurich, Zurich, Switzerland; 4 Institute of Agricultural Sciences (IAS), ETH Zurich, Zurich, Switzerland; 5 Department of Environmental Sciences (DUW), University of Basel, Basel, Switzerland; 6 Institute of Evolutionary Medicine, University of Zurich, Zurich, Switzerland

**Keywords:** Genomics, Genetic techniques, Genomic analysis, Sequencing, Conservation biology, Biological techniques, Genetics, Environmental sciences

## Abstract

The millions of specimens stored in entomological collections provide a unique opportunity to study historical insect diversity. Current technologies allow to sequence entire genomes of historical specimens and estimate past genetic diversity of present-day endangered species, advancing our understanding of anthropogenic impact on genetic diversity and enabling the implementation of conservation strategies. A limiting challenge is the extraction of historical DNA (hDNA) of adequate quality for sequencing platforms. We tested four hDNA extraction protocols on five body parts of pinned false heath fritillary butterflies, *Melitaea diamina*, aiming to minimise specimen damage, preserve their scientific value to the collections, and maximise DNA quality and yield for whole-genome re-sequencing. We developed a very effective approach that successfully recovers hDNA appropriate for short-read sequencing from a single leg of pinned specimens using silica-based DNA extraction columns and an extraction buffer that includes SDS, Tris, Proteinase K, EDTA, NaCl, PTB, and DTT. We observed substantial variation in the ratio of nuclear to mitochondrial DNA in extractions from different tissues, indicating that optimal tissue choice depends on project aims and anticipated downstream analyses. We found that sufficient DNA for whole genome re-sequencing can reliably be extracted from a single leg, opening the possibility to monitor changes in genetic diversity maintaining the scientific value of specimens while supporting current and future conservation strategies.

## Introduction

Natural history collections host approximately two million species represented by three billion individual specimens^1,2^. These specimens are meticulously collected, preserved, and curated and represent all major taxonomic groups from across the globe. The specimens and associated metadata are invaluable for a wide array of research including ecological and environmental research, systematics, and taxonomy^1,3^. Historical DNA (hDNA) from such specimens can nowadays be sequenced using next-generation short read sequencing technologies^4^, allowing the analysis of entire genomes up to several centuries old^5^. Pinned specimens from museums and entomological collections are a unique and invaluable resource for studying the genetic diversity at the species and population level^6,7^, applying molecular methods on specimens from the past^8,9^ and extinction dynamics^10^.

Extracting DNA from historical insect specimens presents a unique set of challenges compared to newly collected individuals. Shortly after the death of the organism, DNA strands progressively break down due to biological processes, such as enzymes from the organism itself^11^ and chemical damage, e.g. hydrolysis and oxidation^12^. Pinned specimens that are kept in collections are largely protected from major degrading agents in the natural environment (bacteria and fungi) with little chemical fumigation, but they are not sterile and usually kept at room temperature, which allows natural DNA degradation to continue and the colonisation by microorganisms. DNA extracted from such specimens is thus only present in small quantities, is heavily degraded, and is subject to environmental contamination of various sources/origins. Thus, the molecular nature of hDNA necessitates specific laboratory procedures, sequencing strategies, and analysis techniques^13^.

Since the success of DNA isolation depends on the ability to remove or bind inhibitory compounds, either from the processed biological sample (e.g. chitin, phenolic compounds) or the chemical of choice (e.g. DTT, PTB, or CTAB) for making the DNA recoverable^14^, choosing the most efficient extraction buffer is a crucial step for the extraction of DNA of sufficient quality and quantity from both modern and in particular historical specimens. Although dozens of different extraction protocols have been published, few studies have investigated which one is most efficient for a specific taxonomic group, type of tissue, or preservation type^15,16^. Published studies for pinned and historic insects use a variety of protocols, sometimes without providing any additional rationale for the protocol selection beyond the shared use of using historic/ancient material^14,17–18^, despite indications that DNA recovery from historical material might be enhanced by carefully choosing the extraction buffers^20^. Unfortunately, common DNA extraction methods are inherently destructive for the specimen, as it either involves crushing the entire sample or removing a single appendage^20,22,23^. This is an obvious caveat in the case of rare and valuable specimens, and it is in the interest of both curators and researchers to minimise damage during sampling. For this reason, some non-destructive or semi-destructive protocols have been developed in recent years to extract hDNA with minimal damage, mostly based on full immersion of the specimen or abdomen and then recovery and re-pinning^14,19,24,25^, but such procedures mostly do not allow for a second DNA extraction and are very labour-intensive.

Lepidoptera is the second-most diverse (by extant species described) and best-known order of insects^26^. Butterflies and moths are highly valuable for monitoring biodiversity^27^, and since insects have suffered major die-offs over recent decades^28^, unravelling past genetic diversity levels of Lepidopterans is of major ecological and conservation interest^8,9^. However, due to their fragile morphology, in particular of wings and other extremities, members of the order Lepidoptera (butterflies and moths) are not well suited for non-destructive DNA extraction methods, mainly because their wings do not allow for total immersion of the insect in lysis buffers. Instead, removal and use of individual legs of a butterfly is a widely accepted procedure by curators because it preserves the integrity of wings and other structures important for species identification. Nevertheless, due to their limited biomass, Lepidopteran legs may yield smaller amounts of DNA than other body parts. Unfortunately, while differences among body parts as sources of hDNA have been explored for vertebrates^29–31^, to our knowledge, the evaluation of this relationship for pinned insects remains largely undiscussed. Further, it is known that different tissue types contain different densities of mitochondria^32^, with particularly high densities of mitochondria in insect flight muscles, due to their high energy demand^33^. This aspect can be of interest depending on the scope of a study.

To our knowledge, there are not many protocols specifically designed for the extraction of hDNA for whole-genome re-sequencing (WGS) of Lepidoptera^34^, and many studies use Targeted Enrichment (TE) approaches, reducing the cost of large phylogenomics studies^35,36^. Analysing mitochondrial DNA (mtDNA) can be another cost-efficient but powerful approach, e.g., to conduct phylogenetic analyses and trace maternal lineages. Whole-genome re-sequencing uses mostly nuclear DNA (nuDNA) and retrieves more information and opening far more possibilities for analysis and inference^4^. We believe that researchers should aim for WGS methods to make the best use of precious historical specimens. This allows using the gained data for multiple purposes, from inferences of adaptive and neutral genetic diversity to phylogeography and phylogenetics, as well as genome scans and temporal genomics. However, the DNA needs to contain as little contamination of foreign (non-target species) DNA as possible and contain as much nuDNA as possible compared to mtDNA to keep sequencing costs low. Nowadays, most collections understand themselves not only as archives of past and current organisms but also as active providers of samples for molecular research. Samples from collections are precious, and it is the researchers’ responsibility to obtain the greatest possible information content from a single specimen with as little damage as possible.

In this study, we tested four different hDNA extraction protocols as well as different body parts—head, thorax, abdomen, wings, and legs—to determine the most effective combination for extracting and sequencing hDNA from pinned specimens of Lepidoptera (i.e., *Melitaea diamina*) suitable for WGS analyses while preserving the specimens' physical integrity. Furthermore, we investigated the efficiency and usefulness of various body parts in delivering nuDNA or mtDNA sequencing data, as well as the success rate and quality of extracted DNA, as this is critical for recommending specific protocols based on the collection curator's material and whether the research question is answered by analyzing nuDNA or mtDNA. We identified a buffer that works with only a single leg and yields the best nuDNA to mtDNA ratio, affecting the Lepidoptera specimen the least and providing enough endogenous DNA for WGS sequencing.

## Material and methods

### Specimen selection and collection procedure

We used nine pinned false heath fritillary butterflies (*Melitaea diamina* (Lang, 1789); synonym *M. dyctinna*), ranging in age from 60 to approximately 100 years old, which were donated by the Entomological Collection of ETH Zurich, Switzerland (Table 1). *M. diamina* reaches a wingspan of about 33 mm, and has the typical wing markings of their genus, which are distinctively darkened on the hind wings in this species. The species is listed as near threatened (NT) on the Swiss Red List. Its distribution is patchy, and it can be found in both damp and sunny environments, such as litter meadows on the edge of bogs and fens, as well as forests that receive a lot of moisture and light^37^.

**Table 1:** Specimens used in this study, with the correspondent ID from the Entomological Collection of ETH Zurich.

Collection ID	Date	Sample ID
Drawer 4/2, *Melitaea dyctinna*, Helvetia 195 No 1*	1873–1968	Mdi_BERGU_TH01
Drawer 4/2, *Melitaea dyctinna*, Helvetia 195 No 2*	1873–1968	Mdi_UNK_TH02
Drawer 4/2, *Melitaea dyctinna*, Helvetia 195 No 3*	1873–1968	Mdi_UNK_TH03
Drawer 4/2, *Melitaea dyctinna*, Helvetia 195 No 4*	1873–1968	Mdi_UNK_TH04
Drawer 49, *Melitaea dyctinna*, No 1†	1840–1920	Mdi_UNK_TH05
Drawer 49, *Melitaea dyctinna*, No 2†	1840–1920	Mdi_UNK_TH06
Drawer 49, *Melitaea dyctinna*, No 3†	1840–1920	Mdi_UNK_TH07
ETHZ-ENT0033345 **	1928	Mdi_UNK_TH08
ETHZ-ENT0033409***	1962	Mdi_WILLE_TH01

*Samples collected by Fritz Carpentier (1873–1968) at an unknown exact date and thus lack the regular ETH-identifier.

^†^Samples collected by Gustav Huguenin (1840–1920) at an unknown exact date and thus lack the regular ETH-identifier.

**Collected in Bergün, Grisons, Switzerland.

***Collected in 1962 in Willerzell, Schwyz, Switzerland.

**Figure 1:**
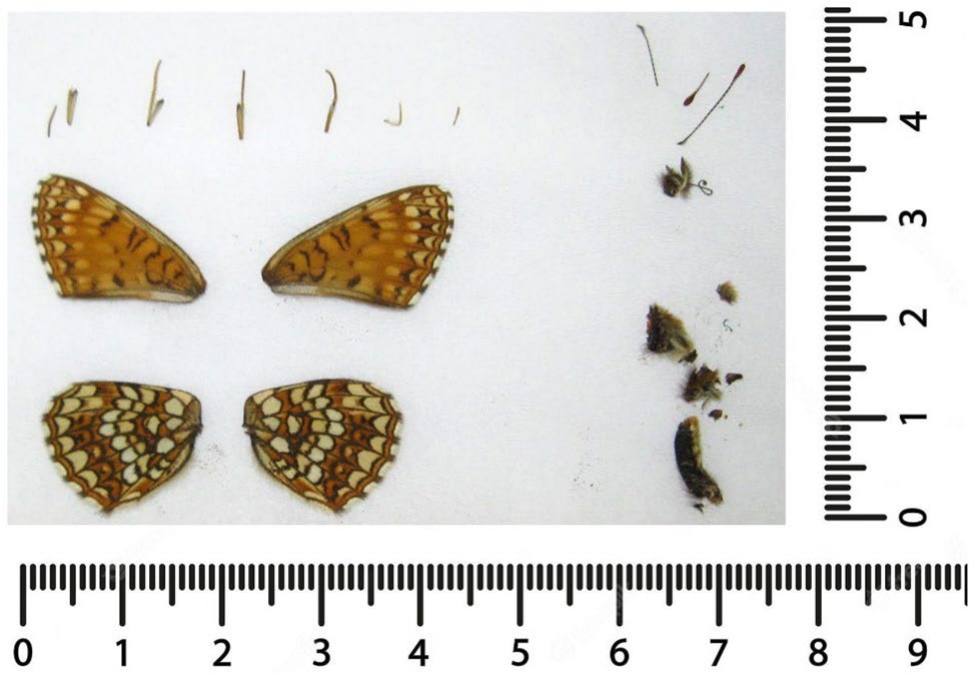
*Melitaea diamina* specimen removed from its collection pin, dismembered into the five different body parts (legs, head, wing, thorax, and abdomen), and laid out for documentation before being collected in individual tubes for DNA extraction. Digital scale in centimetres (cm).

We designed a balanced test set by separating the five different body parts of each individual specimen (legs, head, wing, thorax, and abdomen) for testing tissue type related hDNA extraction buffer efficiency. In order to prevent contamination from other specimens and from fresh human DNA, the entire specimen was collected directly at the Entomological Collection of ETH Zurich following gold-standard recommendations from the ancient DNA field^4,11,14^ (use of full-body protective suits, cleaning of surfaces with DNA AWAY®, Carl Roth Gmbh + Co. KG, Germany, and UV light, use of single use equipment such as gloves to avoid cross-contamination) and transferred in clean containers to the cleanroom facilities, which are dedicated solely to aDNA/hDNA research at the Institute of Evolutionary Medicine, University of Zurich. Each specimen was carefully dismembered with decontaminated tweezers and separated into the following main body parts: head, thorax, wings, legs, and abdomen (Fig. 1). Each body part was then placed in a 2 mL Eppendorf collection tube, ready for extraction, resulting in a total of 115 subsampled body parts from which 68 were processed (Supplementary Information; Table S1, Extended Sample List).

### DNA extraction and library preparation

We selected four different extraction buffers based on hDNA application, proven efficiency, and relative simplicity. *Buffer 1*^14^ is composed of EDTA, Proteinase K, Tris, NaCl, CaCl2, and DTT. *Buffer 2*^38^ contains GuSCN, *β* -mercaptoethanol, Tween, Tris, EDTA, and NaCl. *Buffer 3*^39^ contains SDS, Tris, Proteinase K, EDTA, NaCl, PTB, and DTT. Finally, *Buffer 4*^40^ contains CTAB, Tris–HCl, EDTA, NaCl, *β*-mercaptoethanol, and SDS (Table 2). Detailed information can be found in the Supplementary Information (Table S2; Extraction Buffer Composition). For a better understanding of the extraction protocols, we give a brief explanation of the main reagents used in the different buffers. EDTA (C_10_H_16_N_2_O_8_) chelates divalent cations and causes cell wall rupture to release the nucleic acids to the medium^41^; *β*-mercaptoethanol acts as a strong reducing agent of phenolic compounds, such as tannins and other polyphenols, e.g. from plant extracts; but *β*-mercaptoethanol also reduces disulfide bonds of proteins, preventing DNA cross-linkage^18^. Tris equilibrates the pH to be close to 8.0, optimal for most lysis. Sodium chloride (NaCl) aids in the extraction of nucleic acids from polysaccharides and the removal of proteins that have become cross-linked to DNA^42^. CTAB (hexadecyltrimethylammonium bromide) is a cationic detergent that captures the lipids and complex polysaccharides that can co-precipitate with the DNA^43^. N-phenacyl thiazolium bromide (PTB) also cleaves glucose-derived protein crosslinks, and it can help to release DNA trapped within sugar-derived condensation products^44^; it is widely used in archaeobotanical protocols^39,45^. In total, each Buffer extracted a total of 17 body parts and a negative control that consisted only of the reagents to monitor external and cross-contamination.

**Table 2:** Composition of each of the hDNA extraction buffers used in the present study.

*Buffer 1*^14^	*Buffer 2*^37^	*Buffer 3*^38^	*Buffer 4*^39^
EDTA (0.5 M)	EDTA (0.5 M)	EDTA (0.5 M)	EDTA (0.5 M)
Tris pH 8 (1 M)	Tris pH 8 (1 M)	Tris pH 8 (1 M)	Tris–HCl (1 M)
NaCl (5 M)	NaCl (5 M)	NaCl (5 M)	NaCl (5 M)
Proteinase K (0.4 mg/mL)	–	Proteinase K (0.4 mg/mL)	–
–	B-mercaptoethanol (1%)	–	B-mercaptoethanol (1%)
DTT (40 mM)	–	DTT (40 mM)	–
	GuSCN (5 M)	–	–
CaCl2 (110.98 g/mol)	–	–	–
–	Tween (1%)	–	–
–	–	PTB (2.5 mM)	–
–	–		CTAB 10%
–	–	SDS (1%)	SDS (20%)

**Figure 2:**
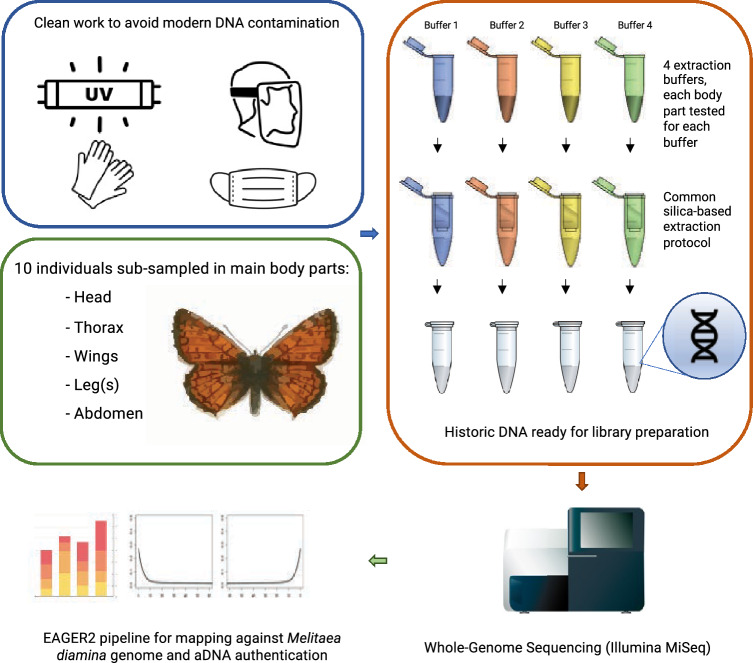
Overview of the workflow of this study including sample collection, sample preparation, DNA extraction and sequencing and bioinformatic analysis of whole-genome re-sequencing historical DNA data.

For every buffer and body part, a total volume of 1.2 mL was added to a 2 mL collection tube, sealed with paraffin, and incubated overnight (circa 16 h) on a nutating mixer (Corning® LSE™, US) at 37 °C. After digestion, samples were extracted following a silica-based extraction protocol as described by Dabney and colleagues^46^ (Fig. 2) with modifications: the binding buffer volume was increased to 15 mL, and the QIAquick spin columns (QIAGEN, Germany) had attached Zymo-Spin V funnels (Zymo Research, Germany) that were bleached and UV irradiated prior use; the combined column and funnel were introduced into 50 mL centrifuge-safe falcon tubes to allow the flowthrough of the higher amount of buffer.

After successful extraction of hDNA, double-stranded DNA libraries were generated following a well-established, non-commercial protocol designed to efficiently blunt-end repair and sequence ultrashort DNA fragments characteristic of aDNA^13^. All PCR amplification, post-PCR, and next-generation-sequencing (NGS) analyses were carried out in physically separated laboratories, and negative controls containing only reagents were added both during DNA extraction and library construction to control for contamination events, a total of 7 blanks—four Extraction Blanks and three Library Blanks. All 68 libraries and 7 blanks were pooled aiming for equimolarity of the samples (molarity measured with Tapestation, Agilent Technologies) and 150 bp paired-end sequenced on an Illumina MiSeq™ system at the Genetic Diversity Centre (GDC), ETH Zurich. However, because of the nature of the short hDNA fragments and library insert size, the forward reads were only sequenced for 100 bp (cycles) to avoid the risk of a sequencing abort of the MiSeq and loss of the individual index readout. The reverse read was intended to be sequenced for all 150 cycles but aborted after 28 cycles due to a technical malfunction of the Illumina MiSeq machine. This, however, did not impair our results as merging of forward and reverse reads was feasible, as 11 bp is the default minimum required overlap and the average hDNA fragment length is usually far below 100 bp, in this study as well.

### Sequencing data processing and analysis

To assess the sequencing data, we used a pipeline based on the PALEOMIX BAM-pipeline^47^. Briefly, forward and reverse reads were adapter-clipped and merged using AdapterRemoval v2.3.3^48^ with the settings *–mm 3 –minlength 25 –collapse-conservatively –trimns –trimqualities*, requiring a default minimum overlap of 11 bp. Reads were aligned with bwa-mem 2^49^ against both mitochondrial genome and the draft de novo* M. diamina* reference genome (butterfly_v1.asm.bp.p_ctg_mtDNA_masked.fa)^49,50^. We filtered mapped reads for a minimum mapping quality of Q20 using sambamba v0.8.0^50^, then removed PCR-duplicates with picardTools v2.27.5 *MarkDuplicates* (http://broadinstitute.github.io/picard/) and obtained mapping statistics with ATLAS *BAMDiagnostics*^51^. An important characteristic of ancient sequencing libraries is the occurrence of deamination, the transition of C to T bases at 5’ ends, and G to A at 3’ ends of DNA fragments, which is a signature used to authenticate hDNA^12^. We used mapDamage v2.0^52^ with default settings to estimate deamination at 5’ and 3’ ends for ancient and historic DNA authentication. Fragment size distributions and misincorporation rates were estimated, validated, and compared at the level of individuals by merging BAM-files that were generated for individual body parts to obtain one file per individual to improve the accuracy of misincorporation rate estimations. The draft de novo* M. diamina* reference genome (butterfly_v1.asm.bp.p_ctg_mtDNA_masked.fa) was based on 6.2 Gb PacBio HiFi reads and assembled and sequenced at the Functional Genomics Center Zurich, FGCZ. The genome was assembled and purged for duplicates with hifiasm^53^ and the parameters -l3 -s 0.55. The assembled genome is 805 Mb long and encompasses 3,918 contigs and has a BUSCO (v5.2.2 arthropoda_odb10;^54^) value of 96.2%.

We analysed yield (number of reads), endogenous content (fraction of reads mapped ≥ Q20) to the reference genome, and nuDNA/mtDNA base count ratios (number of bases mapped to the nuclear genome divided by the number of bases mapped to the mitochondrial genome) in response to the buffer and body part used with ANOVA and TukeyHSD-tests in R v4.2.1^55^. To meet normality assumptions, nuDNA/mtDNA ratios were log-transformed for statistical testing. For hDNA authentication and summaries at the level of individuals we combined reads from all body parts obtained from the same individual (Supplementary Table, Sheet 1). Due to very low endogenous DNA content, we excluded one individual (Mdi_UNK_TH04) and all eight associated body part extractions from all further analyses. For analyses of effects of body parts, we used for each individual the averaged values over all leg samples to avoid pseudo replication, resulting in 8 extra in silico generated samples added (Supplementary Table, Sheets 2 & 3).

## Results

### Sequencing output and endogenous content

**Figure 3:**
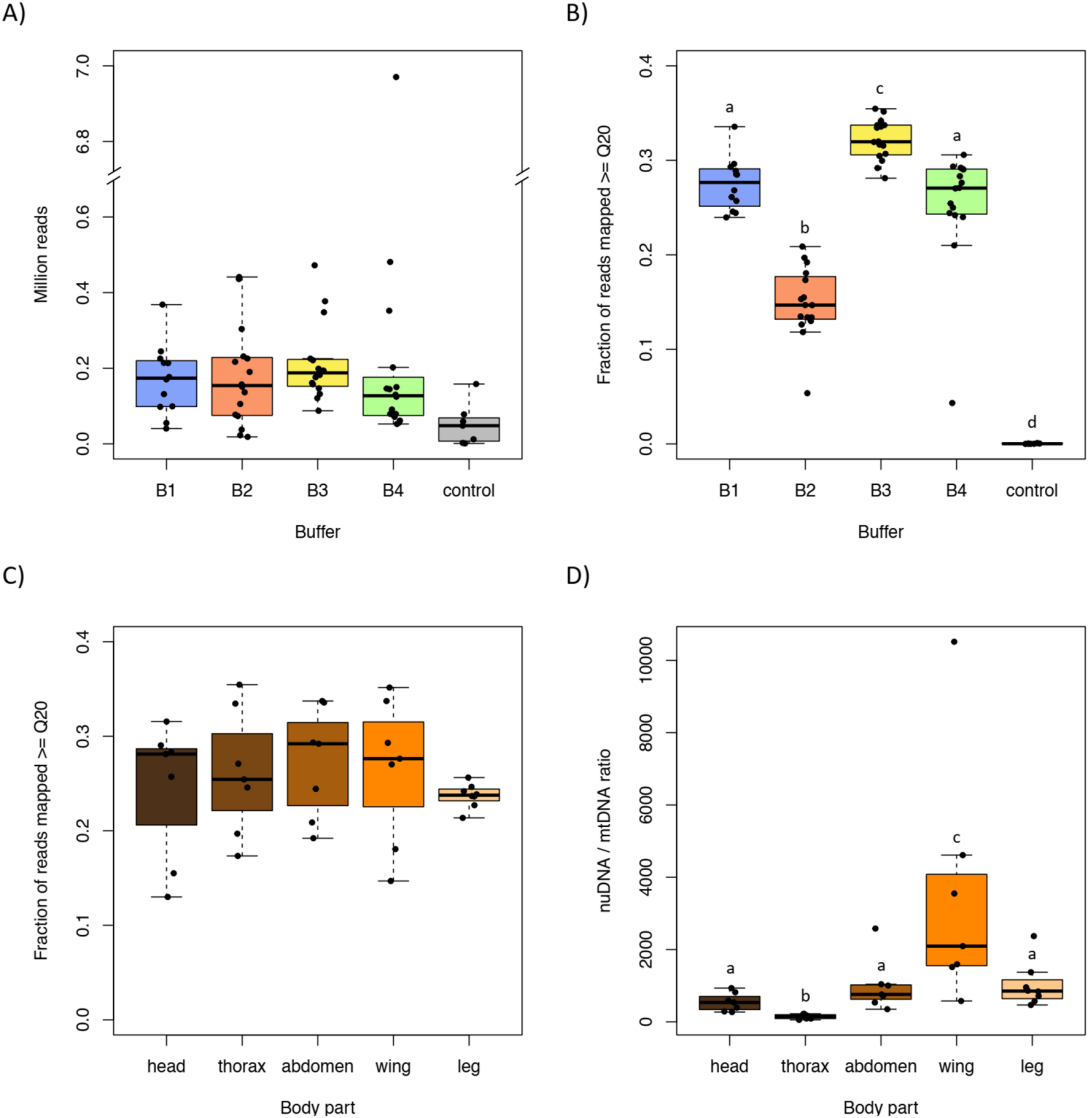
(**A**) The total number of sequenced reads per sample, divided into groups based on the type of hDNA extraction buffer (B1, B2, B3 and B4) used, and a collection of negative blank reads (control). (**B**) Fraction of reads mapped to the *Melitaea diamina* reference genome with a mapping quality > Q20 (endogenous content), separated by buffer type, with negative blanks combined. (**C**) The fraction of reads mapped to the *M. diamina* reference genome (> Q20, endogenous content), separated by body part of the sample. (**D**) The ratio of bases mapped to the nuclear vs mitochondrial *M. diamina* reference genome. Higher values indicate more bases mapping to the nuclear genome (nuDNA) relative to the number of bases mapping to the mitochondrial genome (mtDNA).

Overall, we obtained 19.4 million reads in this shallow Illumina MiSeq sequencing run, with an average read numbers of 2.1 million per individual, ranging from 0.76 to 8.0 million reads. Our forward reads were limited to 100 bp and our reverse reads 28 bp, due to a technical malfunction of the Illumina MiSeq machine, resulting in a maximum insert size of merged reads of 117 bp (100 bp forward + 28 bp reverse—11 bp default overlap, see Material and Methods). We only processed successfully merged reads (93.5% of all reads). Reads outside the range of 30 bp–117 bp are not mapped and do not contribute to our fragment length statistics. The DNA yield (i.e. number of merged reads) did not differ significantly between the different hDNA extraction buffers (P-value for all pairwise comparisons > 0.05; Fig. 3A), an innate observation, because all the produced sequenced libraries were pooled equimolarly based on a qPCR to normalize the sequencing output. Nonetheless, one single sample did yield significantly more reads than any other sample (Fig. 3A, Mdi_UNK_TH03, abdomen, see Supplementary Table, Sheet 1), likely due to a pipetting error during the pooling that led to over loading. Since the number of reads does not indicate successful extraction and sequencing of endogenous hDNA, we mapped merged reads to the *Melitaea* reference genome and expressed the endogenous DNA content as the percentage of successfully mapped reads with a mapping quality ≥ Q20 (Fig. 3B). Combining the reads per individual, endogenous and uniquely mapped reads content varied between individuals from 20.5% to 31.6%, except for one specimen (Mdi_UNK_TH04, all body parts, see Supplementary Table, Sheet 1) that had for all eight body parts an endogenous DNA content of less than 0.25%, regardless of the buffer used. Therefore, the failure of this sample was specimen-specific and was excluded from all statistical analyses, as were the blanks (Supplementary Table, Sheet 1). Coverage on the level of the remaining eight individuals ranged from 0.01 – 0.14x. Endogenous content differed significantly between hDNA extraction buffers (Fig. 3B), with the highest value for *buffer 3* (mean 32.2% ± 2.1% SD), which differed significantly from *buffer 1* (P = 0.02; mean 27.5% ± 2.8% SD) and *buffer 4* (P < 0.001; mean 25.4% ± 6.2% SD). Endogenous content was significantly lower for samples extracted with *buffer 2* (mean 14.9% ± 3.7% SD) compared to the other three buffers (P < 0.001 in each case). Out of the 76 samples analysed, 44 had > 20% endogenous content and 15 had endogenous contents > 30%. On the lower end, 10 samples and all 7 blanks sequenced for control had an endogenous content < 0.1% (eight from the excluded individual and two additional leg samples) and 14 samples had variable endogenous contents between 0.1% and 20% (Supplementary Table, Sheet 2). Endogenous content did not differ significantly between body parts (Fig. 3C; P > 0.05 for each comparison, with means of 27.2% ± 8.5% SD for abdomens, 24.5% ± 7.2, 23.7% ± 1.3% for heads, 26.2% ± 6.6% for legs and 26.5 ± 7.6% for wings).

### Nuclear and mitochondrial DNA content

The nuDNA/mtDNA base count ratio however, differed significantly between body parts (Fig. 3D), with the highest nuDNA/mtDNA ratio found in wings (mean ratio of 3495 ± 3280 SD, all comparisons with other body parts P < 0.05). DNA extractions from legs, abdomens, and heads did not differ significantly from one-another in terms of nuDNA/mtDNA ratios (P > 0.05 for each comparison with mean 1019 ± 611 SD for legs, 998 ± 740 SD for abdomens and 547 ± 256 SD for heads). The lowest nuDNA/mtDNA ratios were observed in extractions from thoraxes (P < 0.01 for each comparison mean 144 ± 67 SD).

### Historical DNA authentication

**Figure 4:**
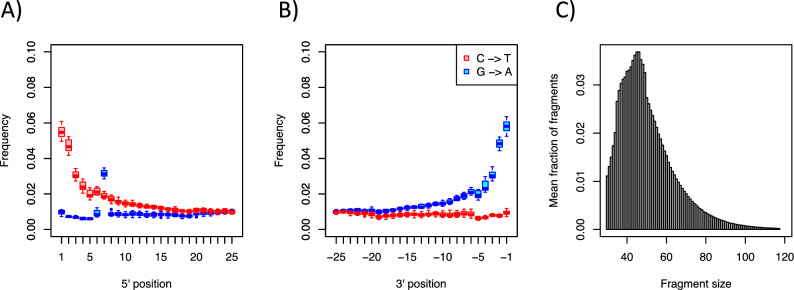
Ancient DNA damage patterns for eight *Melitaea diamina* samples mapped to the reference genome. Base misincorporation frequency at (**A**) 5’ and (**B**) 3’ read ends, summarised as box plots showing C to T and G to A transitions. (**C**) Fragment length distribution summary of reads. Bars show the mean fraction of fragments for each possible fragment size.

Regarding the authenticity of historical DNA, each sample displayed the expected deamination rate of 2–5% in the five outermost base pairs, which is typical of hDNA from insect collections^25,21^, but lower than rates from aDNA samples such as teeth and fossil bones. It confirms that we produced historic DNA libraries (Fig. 4A,B). The observed deamination rate spike at position 6 and 7 (G->A) inside the reads (Fig. 4A) was caused by a technical disturbance during the Illumina MiSeq sequencing and affected all reads equally and should thus not affect our results. The average DNA fragment length per individual ranged from 45.2 bp ± 11.7 SD to 50.7 bp ± 14.0 SD, falling within the typical range for hDNA standards^11^ (Fig. 4C).

## Discussion and conclusions

We aimed to identify the best combination of body part and buffer for the extraction and sequencing of nuclear hDNA from pinned Lepidoptera with minimal impact on their integrity, maintaining their collection value. To achieve this, we conducted a comparative study using four different extraction buffers on five distinct butterfly body parts. While all buffers were successful in extracting authentic hDNA (Fig. 4), there were observable differences in the effectiveness of the sequencing, as shown by the endogenous content (Fig. 3B). *Buffer 3* yielded the highest endogenous DNA content, followed by *buffers 1* and *4*, whilst the lowest endogenous content was observed in *buffer 2*. Thus, from a sequencing perspective, *buffer 3* would be the preferred option for extracting hDNA from pinned insect material. Further, our results show that especially Lepidoptera legs are ideal for retrospective genetic analysis. First, collections are more likely willing to sacrifice a single leg than other bigger structures. And second, legs deliver a high ratio of nuDNA to mtDNA (26.2% ± 6.6% SD; Fig. 3D), which helps to reduce sequencing costs.

The exact composition of the hDNA extraction buffers and their effects on specific body parts could explain why there were significant differences in endogenous DNA extraction efficiency regardless of the type of tissue used. Both EDTA (a chelating agent), and Proteinase K (a subtilisin-related alkaline serine protease), are usually the most used reagents in extraction buffers for ancient and historic DNA protocols^29^. Dithiothreitol (DTT) is also a commonly used reagent, added to aid in protein digestion by reducing sulfide bonds, helping to release thiolated DNA into solution, and possibly reducing cross-links between DNA and other biomolecules^56^. PTB can help release trapped DNA inside sugar-mediated condensation products by cleaving glucose-derived protein crosslinks^57^. Considering that *buffer 3* was mainly based on compounds intended to extract DNA from plant material^39^, the reagents' intended effect on insoluble carbohydrates from plant tissue is also extremely effective on chitinous insect tissue. The combined effect of proteinase K and DTT could explain why DNA extraction from body parts with sclerotin is more efficient. Nitrogen-rich polysaccharides of chitin are cross-linked to proteins and phenolic compounds, and more importantly in the current case, free DNA from the insect cells liberated after cell death in the tissue. We thus claim that *buffer 3* is better than the others because it can release a higher amount of endogenous insect hDNA that has been sequestered by chemical compounds from the sclerotin-rich tissue of insects. This effect increases the proportion of endogenous DNA compared to exogenous DNA from other sources present in the sample, such as bacteria and fungi.

Although we saw variations in endogenous content between hDNA extraction buffers, there were no appreciable variations in yield, as shown by the quantity of reads obtained. Since the libraries were PCR-amplified and pooled equimolarly prior to sequencing^13^, we did anticipate that, under similar conditions, the number of sequencing reads obtained from each library would be roughly equal. Opposed to other sequencing criteria like read yield, endogenous content is thus a more useful metric to evaluate the effectiveness of the buffers, as it realistically captures the ratio of specimen DNA to foreign DNA (contamination, microbial) in our samples. Our results presented here indicate that the butterfly leg is a reliable source of endogenous DNA similar or even better than other body parts.

As a result, legs are a desirable tissue for sampling hDNA from Lepidoptera, and, presumably, also for many other insects or Arthropoda taxa with comparable-size legs^20,22,58^. The structure of the legs, as tubular outgrowths of the body wall filled with haemolymph, provides a straightforward and symmetrical anatomical feature for easy sampling and preservation. Sampling symmetrical or repeated features such as legs preserves anatomical information in the form of the mirrored body part. Furthermore, removing larger structures like wings, abdomen, or head usually compromises the specimen's integrity based on our experience. Removing only the thorax without disassembling the specimen is impossible, and often results in the shattering of the thorax. Collecting only one leg takes advantage of symmetrical features, preserves tissue for possible future sampling (e.g., remaining legs) and ensures integrity of structures relevant for species identification such as wings. Since the leg is smaller than other insect body parts, it can be fully submerged in the extraction buffer and recovered if necessary. In fact, the higher buffer-to-body-part ratio may have aided DNA recovery. Additionally, legs often detach from pinned insects by accident, making them ideal for molecular research if they can be assigned to a specific specimen. From a molecular preservation point of view, the lower mass/surface ratio of legs could facilitate rapid drying, thus minimising DNA damage from hydrolysis and improving preservation. In addition, since most of the tissue in the leg is of chitinous/sclerotic nature, the cross-linking effect mentioned above between DNA and polysaccharides could ensure DNA binding and facilitate preservation even further. With similar explanations, recent investigations have also chosen to harvest DNA from ancient insect specimens using their legs^22,58^, although without prior testing of body parts or buffer as is the case in the present study.

Tissue-dependent variations in DNA concentration, such as differences in nuDNA/mtDNA content among different insect tissues, may also be considered when selecting a body part to sample. High rates of nuDNA are desirable when WGS is performed, as sequencing excessive amounts of mtDNA increases sequencing effort and hence costs without providing additional information on the nuclear genome. Our results show low amounts of mtDNA relative to nuDNA in wings and legs, adding to the desirability of legs as sampling tissue for WGS projects. The nuDNA/mtDNA ratio is about seven-fold higher in legs compared to thoraxes, meaning a seven-fold lower loss of reads to mtDNA when using legs for WGS instead of thoraxes. The low amount of mtDNA in legs and wings may be explained by the fact that they don’t contain large amounts of muscles, but instead their cavities are filled with nuclei-containing haemolymph and connective tissue^59^. Thoraxes on the other hand had the highest amounts of mtDNA. In butterflies, the muscles for wing movement are located in the thorax^59^, and the need to maintaining high metabolic rates over extended periods of time results in a higher concentration of mitochondria in thorax tissues than in other sections of the body^60^. However, despite the strong differences in nuDNA/mtDNA ratio between tissue types, this difference plays a rather minor role in molecular practice, as the number of bases “lost” to the mtDNA, is only about 0.7% across different body parts.

Our optimised extraction method, applied to a single leg cut or broken from the specimen, provides a practical approach to minimise damage while obtaining sufficient DNA for downstream WGS analyses. The amount of useful data generated for genetic diversity estimations is remarkable considering the little involved input provided by a low-mass body part as is the butterfly leg. The combination of a high-chitinous tissue with a hDNA extraction buffer that effectively dissolves polysaccharides likely ensured the retrieval of hDNA with minimum input material and low losses to mtDNA sequencing. The use of this minimally invasive extraction method can have a significant impact on the genetic analysis of valuable collection specimens and provide critical insights into their evolutionary history without compromising the integrity of the specimen. Nonetheless, further studies are necessary to assess the applicability of our method to additional taxa and specimen types within and beyond Lepidoptera. The fact that our method worked as satisfactory with such minimal starting material and a common tissue type among most insect species—a single leg—suggests that it will likely work well with a variety of pinned specimens since legs of butterflies follow a relatively standard structure and composition when it comes to insect anatomy.

### Supplementary Information

Supplementary Information 1.

Supplementary Information 2.

## Data Availability

The raw MiSeq reads and PacBio HiFi long reads for the *Melitaea diamina* individuals can be found on the NCBI Sequence Read Archive (SRA, PRJNA1106412). The *M. diamina* reference genome assembly (butterfly_v1.asm.bp.p_ctg_mtDNA_masked.fa) is available at the Dryad digital repository (10.5061/dryad.pzgmsbcvf. Scripts for the bioinformatics and statistical analysis are available upon request to the authors.
