# Supplementary material for: Minimally destructive hDNA extraction method for retrospective genetics of pinned historical Lepidoptera specimens
Source: Sci Rep. 2024 Jun 5;14:12875. doi: 10.1038/s41598-024-63587-7 (PMC11150399; doi:10.1038/s41598-024-63587-7)
Supplement: Supplementary file 1 — Supplementary Information 1. [file 41598_2024_63587_MOESM1_ESM.docx]

**Supplementary Information**

**Minimally destructive hDNA extraction method for retrospective genetics of pinned historical Lepidoptera specimens**

Enrique Rayo^1,2^ (ORCID: 0000-0002-4497-4339), Gabriel F. Ulrich^1^ (ORCID: 0000-0002-2092-1152), Niklaus Zemp^3^ (0000-0002-0991-5130), Michael Greeff^4^ (0000-0002-2697-6330), Verena J. Schuenemann^5,6^ (ORCID: 0000-0002-8593-3672), Alex Widmer^1^ (ORCID: 0000-0001-8253-5137) and Martin C. Fischer^1^* (ORCID: 0000-0002-1888-1809).

_1_ Institute of Integrative Biology (IBZ), ETH Zurich, Zurich, Switzerland

^2^ Institut für Veterinärpathologie, University of Zurich, Zurich, Switzerland

^3^ Genetic Diversity Centre (GDC), ETH Zurich, Zurich, Switzerland

^4^ Institute of Agricultural Sciences (IAS), ETH Zurich, Zurich, Switzerland

^5^ Department of Environmental Sciences (DUW), University of Basel, Basel, Switzerland

^6^ Institute of Evolutionary Medicine, University of Zurich, Zurich, Switzerland

* Corresponding Author: Martin C. Fischer ([martin.fischer@usys.ethz.ch](mailto:martin.fischer@usys.ethz.ch))

**Table S1:** Extended sample list of the specimens used in this study, with the correspondent ID from the Entomological Collection of ETH Zurich. NA on the ‘Weight’ column corresponds to ‘Too low to measure’ given the scale error (0,0001g).

| **Original ID** | **Body part** | **Weight** | **Collection** |
| --- | --- | --- | --- |
| Drawer 4/2, Melitea dyctinna, Helvetia 195 No 1, leg 1 | leg | 0,0007 | Zurich |
| Drawer 4/2, Melitea dyctinna, Helvetia 195 No 1, leg 2 | leg | 0,0007 | Zurich |
| Drawer 4/2, Melitea dyctinna, Helvetia 195 No 1, leg 3 | leg | 0,0007 | Zurich |
| Drawer 4/2, Melitea dyctinna, Helvetia 195 No 1, leg 4 | leg | 0,001 | Zurich |
| Drawer 4/2, Melitea dyctinna, Helvetia 195 No 1, head | head | 0,002 | Zurich |
| Drawer 4/2, Melitea dyctinna, Helvetia 195 No 1, thorax | thorax | 0,006 | Zurich |
| Drawer 4/2, Melitea dyctinna, Helvetia 195 No 1, abdomen | abdomen | 0,009 | Zurich |
| Drawer 4/2, Melitea dyctinna, Helvetia 195 No 1, left upper wing | wing | 0,0016 | Zurich |
| Drawer 4/2, Melitea dyctinna, Helvetia 195 No 1, right upper wing | wing | 0,0013 | Zurich |
| Drawer 4/2, Melitea dyctinna, Helvetia 195 No 1, left lower wing | wing | 0,001 | Zurich |
| Drawer 4/2, Melitea dyctinna, Helvetia 195 No 1, right lower wing | wing | 0,001 | Zurich |
| Drawer 4/2, Melitaea dyctinna, helvetia No 2, leg 1 | leg | NA | Zurich |
| Drawer 4/2, Melitaea dyctinna, helvetia No 2, leg 2 | leg | NA | Zurich |
| Drawer 4/2, Melitaea dyctinna, helvetia No 2, leg 3 | leg | NA | Zurich |
| Drawer 4/2, Melitaea dyctinna, helvetia No 2, leg 4 | leg | NA | Zurich |
| Drawer 4/2, Melitaea dyctinna, helvetia No 2, leg 5 | leg | NA | Zurich |
| Drawer 4/2, Melitaea dyctinna, helvetia No 2, leg 6 | leg | NA | Zurich |
| Drawer 4/2, Melitaea dyctinna, helvetia No 2, left upper wing | wing | NA | Zurich |
| Drawer 4/2, Melitaea dyctinna, helvetia No 2, right upper wing | wing | 0,0004 | Zurich |
| Drawer 4/2, Melitaea dyctinna, helvetia No 2, left lower wing | wing | 0,0008 | Zurich |
| Drawer 4/2, Melitaea dyctinna, Helvetia No 2, right lower wing | wing | 0,001 | Zurich |
| Drawer 4/2, Melitaea dyctinna, Helvetia No 2, head | head | NA | Zurich |
| Drawer 4/2, Melitaea dyctinna, Helvetia No 2, thorax | thorax | 0,01 | Zurich |
| Drawer 4/2, Melitaea dyctinna, Helvetia No 2, abdomen | abdomen | 0,03 | Zurich |
| Drawer 4/2, Melitaea dyctinna, No 3, leg 1 | leg | NA | Zurich |
| Drawer 4/2, Melitaea dyctinna, No 3, leg 2 | leg | 0,0005 | Zurich |
| Drawer 4/2, Melitaea dyctinna, No 3, leg 3 | leg | NA | Zurich |
| Drawer 4/2, Melitaea dyctinna, No 3, leg 4 | leg | 0,0005 | Zurich |
| Drawer 4/2, Melitaea dyctinna, left upper wing | wing | 0,0019 | Zurich |
| Drawer 4/2, Melitaea dyctinna, right upper wing | wing | 0,0015 | Zurich |
| Drawer 4/2, Melitaea dyctinna, left lower wing | wing | 0,0017 | Zurich |
| Drawer 4/2, Melitaea dyctinna, right lower wing | wing | 0,0016 | Zurich |
| Drawer 4/2, Melitaea dyctinna, head | head | 0,0014 | Zurich |
| Drawer 4/2, Melitaea dyctinna, thorax | thorax | 0,007 | Zurich |
| Drawer 4/2, Melitaea dyctinna, No 3, abdomen | abdomen | 0,012 | Zurich |
| Drawer 4/2, Melitaea dyctinna, No 4, leg 1 | leg | 0,0005 | Zurich |
| Drawer 4/2, Melitaea dyctinna, No 4, leg 2 | leg | NA | Zurich |
| Drawer 4/2, Melitaea dyctinna, No 4, leg 3 | leg | NA | Zurich |
| Drawer 4/2, Melitaea dyctinna, No 4, leg 4 | leg | NA | Zurich |
| Drawer 4/2, Melitaea dyctinna, No 4, leg 5 | leg | 0,0005 | Zurich |
| Drawer 4/2, Melitaea dyctinna, No 4, leg 6 | leg | NA | Zurich |
| Drawer 4/2, Melitaea dyctinna, No 4, left upper wing | wing | 0,0019 | Zurich |
| Drawer 4/2, Melitaea dyctinna, No 4, right upper wing | wing | 0,002 | Zurich |
| Drawer 4/2, Melitaea dyctinna, No 4, left lower wing | wing | 0,0017 | Zurich |
| Drawer 4/2, Melitaea dyctinna, No 4, right lower wing | wing | 0,002 | Zurich |
| Drawer 4/2, Melitaea dyctinna, No 4, head | head | 0,0016 | Zurich |
| Drawer 4/2, Melitaea dyctinna, No 4, thorax | thorax | 0,0069 | Zurich |
| Drawer 4/2, Melitaea dyctinna, No 4, abdomen | abdomen | 0,0227 | Zurich |
| Drawer 49, Melitaea dyctinna, No 1, leg 1 | leg | 0,0005 | Zurich |
| Drawer 49, Melitaea dyctinna, No 1, leg 2 | leg | 0,0007 | Zurich |
| Drawer 49, Melitaea dyctinna, No 1, leg 3 | leg | 0,0008 | Zurich |
| Drawer 49, Melitaea dyctinna, No 1, leg 4 | leg | 0,0005 | Zurich |
| Drawer 49, Melitaea dyctinna, No 1, leg 5 | leg | NA | Zurich |
| Drawer 49, Melitaea dyctinna, No 1, leg 6 | leg | 0,001 | Zurich |
| Drawer 49, Melitaea dyctinna, No 1, left upper wing | wing | 0,0018 | Zurich |
| Drawer 49, Melitaea dyctinna, No 1, right upper wing | wing | 0,0019 | Zurich |
| Drawer 49, Melitaea dyctinna, No 1, left lower wing | wing | 0,0022 | Zurich |
| Drawer 49, Melitaea dyctinna, No 1, right lower wing | wing | 0,0023 | Zurich |
| Drawer 49, Melitaea dyctinna, No 1, head | head | 0,0027 | Zurich |
| Drawer 49, Melitaea dyctinna, No 1, thorax | thorax | 0,007 | Zurich |
| Drawer 49, Melitaea dyctinna, No 1, abdomen | abdomen | 0,0089 | Zurich |
| Drawer 49, Melitaea dyctinna, No 2, leg 1 | leg | NA | Zurich |
| Drawer 49, Melitaea dyctinna, No 2, legs 3 and 4 | leg | 0,001 | Zurich |
| Drawer 49, Melitaea dyctinna, No 2, leg 5 | leg | 0,0005 | Zurich |
| Drawer 49, Melitaea dyctinna, No 2, leg 6 | leg | NA | Zurich |
| Drawer 49, Melitaea dyctinna, No 2, left upper wing | wing | 0,0012 | Zurich |
| Drawer 49, Melitaea dyctinna, No 2, right upper wing | wing | 0,0014 | Zurich |
| Drawer 49, Melitaea dyctinna, No 2, left lower wing | wing | 0,001 | Zurich |
| Drawer 49, Melitaea dyctinna, No 2, right lower wing | wing | 0,0012 | Zurich |
| Drawer 49, Melitaea dyctinna, No 2, head | head | 0,0018 | Zurich |
| Drawer 49, Melitaea dyctinna, No 2, thorax | thorax | 0,085 | Zurich |
| Drawer 49, Melitaea dyctinna, No 2, abdomen | abdomen | 0,015 | Zurich |
| Drawer 49, Melitaea dyctinna, No 3, leg 1 | leg | 0,0005 | Zurich |
| Drawer 49, Melitaea dyctinna, No 3, leg 2 | leg | 0,0005 | Zurich |
| Drawer 49, Melitaea dyctinna, No 3, legs 3 and 4 | leg | 0,001 | Zurich |
| Drawer 49, Melitaea dyctinna, No 3, leg 5 | leg | NA | Zurich |
| Drawer 49, Melitaea dyctinna, No 3, right upper wing | wing | 0,0009 | Zurich |
| Drawer 49, Melitaea dyctinna, No 3, left upper wing | wing | 0,0011 | Zurich |
| Drawer 49, Melitaea dyctinna, No 3, right lower wing | wing | 0,0012 | Zurich |
| Drawer 49, Melitaea dyctinna, No 3, left lower wing | wing | 0,001 | Zurich |
| Drawer 49, Melitaea dyctinna, No 3, head | head | 0,0017 | Zurich |
| Drawer 49, Melitaea dyctinna, No 3, thorax | thorax | 0,078 | Zurich |
| Drawer 49, Melitaea dyctinna, No 3, abdomen | abdomen | 0,018 | Zurich |
| Drawer 49, Melitea dyctinna sp No 4, leg 1 | leg | NA | Zurich |
| Drawer 49, Melitea dyctinna sp No 4, leg 2 | leg | NA | Zurich |
| Drawer 49, Melitea dyctinna sp No 4, leg 3 | leg | 0,003 | Zurich |
| Drawer 49, Melitea dyctinna sp No 4, head | head | 0,002 | Zurich |
| Drawer 49, Melitea dyctinna sp No 4, thorax | thorax | 0,006 | Zurich |
| Drawer 49, Melitea dyctinna sp No 4, upper left wing | wing | 0,002 | Zurich |
| Drawer 49, Melitea dyctinna sp No 4, upper right wing | wing | 0,002 | Zurich |
| Drawer 49, Melitea dyctinna sp No 4, lower left wing | wing | 0,001 | Zurich |
| Drawer 49, Melitea dyctinna sp No 4, lower right wing | wing | 0,002 | Zurich |
| Drawer 49, Melitea dyctinna sp No 4, abdomen | abdomen | 0,01 | Zurich |
| ETHZ-ENT0033345, leg 1 | leg | NA | Graubunden |
| ETHZ-ENT0033345, leg 2 | leg | NA | Graubunden |
| ETHZ-ENT0033345, leg 3 | leg | NA | Graubunden |
| ETHZ-ENT0033345, leg 4 | leg | NA | Graubunden |
| ETHZ-ENT0033345, left upper wing | wing | 0,0015 | Graubunden |
| ETHZ-ENT0033345, right upper wing | wing | 0,0014 | Graubunden |
| ETHZ-ENT0033345, left lower wing | wing | 0,0016 | Graubunden |
| ETHZ-ENT0033345, right lower wing | wing | 0,0017 | Graubunden |
| ETHZ-ENT0033345, head | head | 0,0018 | Graubunden |
| ETHZ-ENT0033345, thorax | thorax | 0,0081 | Graubunden |
| ETHZ-ENT0033345, abdomen | abdomen | 0,012 | Graubunden |
| ETHZ-ENT033409, leg 1 | leg | NA | Willerzel |
| ETHZ-ENT033409, legs 2, 3 | leg | NA | Willerzel |
| ETHZ-ENT033409, leg 4 | leg | 0,001 | Willerzel |
| ETHZ-ENT033409, leg 5 | leg | 0,0012 | Willerzel |
| ETHZ-ENT033409, left upper wing | wing | 0,0016 | Willerzel |
| ETHZ-ENT033409, right upper wing | wing | 0,0014 | Willerzel |
| ETHZ-ENT033409, left lower wing | wing | 0,0013 | Willerzel |
| ETHZ-ENT033409, right lower wing | wing | 0,0015 | Willerzel |
| ETHZ-ENT033409, head | head | 0,0029 | Willerzel |
| ETHZ-ENT033409, thorax | thorax | 0,0072 | Willerzel |
| ETHZ-ENT033409, abdomen | abdomen | 0,0056 | Willerzel |

**Table S2:** Extraction Buffer Composition

**Buffer 1 -** Bases on Campos *et al.* (2019)

| **Buffer 1 (final concentration):** | **Per sample (based in our stocks)** |
| --- | --- |
| EDTA (0.5M) (2.5 mM) | 5 uL |
| Prot K (0.4 mg/mL) | 40 uL |
| Tris PH 8 (1M)(10 mM) | 10 uL |
| NaCl (5M) (10 mM) | 2 uL |
| CaCl2 (110.98 g/mol)(5 mM) | 0.55 mg |
| DTT (40 mM) | 6.17 mg |

**Buffer 2 -** Bases on Smith *et al.* (2021)

| **Buffer 2 (final concentration):** | **Per sample (based in our stocks)** |
| --- | --- |
| GuSCN (final 5M) | 0.59 g |
| B-mercaptoethanol (1%) | 10 uL |
| Tween (1%) | 0.01 uL |
| Tris pH8 (1M)(final 50mM) | 500 uL |
| EDTA (0.5M) (20mM) | 40 uL |
| NaCl (5M) (25mM) | 5 uL |

**Buffer 3 -** Based on Gutaker *et al.* (2017)

| **Buffer 3 (final concentration):** | **Per sample (based in our stocks)** |
| --- | --- |
| SDS (1%) | 0.01 g |
| Tris pH8 (10mM) | 10 uL |
| Proteinase K (0.4 mg/mL) | 40 uL |
| EDTA (10mM) | 20 uL |
| NaCl (5mM) | 1 uL |
| PTB (2.5 mM) | 0.71 mg |
| DTT (50 mM) | 7.71 mg |

**Buffer 4 -** Based on Calderón-Cortés *et al.* (2010)

| **Buffer 4 (final concentration). Preparation for 50 mL** |
| --- |
| CTAB 10% 10mL (5mg in 50 mL) |
| Tris-HCl 1M 5mL (in 50mL) |
| EDTA 0.5M 2mL (in 50mL) |
| NaCl 5M 17.5mL (in 50mL) |
| B-mercaptoethanol 3 uL (added after to the total volume) |
| SDS 20% 10 uL (added after to the total volume) |
| H2O – complete to 50 mL |

**References**

Campos, Paula F., and M. Thomas P. Gilbert. "DNA extraction from keratin and chitin." Ancient DNA: Methods and protocols (2019): 57-63.

Smith, Aaron D., Marcin J. Kamiński, Kojun Kanda, Andrew D. Sweet, Julio L. Betancourt, Camille A. Holmgren, Elisabeth Hempel, Federica Alberti, and Michael Hofreiter. "Recovery and analysis of ancient beetle DNA from subfossil packrat middens using high-throughput sequencing." Scientific Reports 11, no. 1 (2021): 12635.

Gutaker, Rafal M., Ella Reiter, Anja Furtwängler, Verena J. Schuenemann, and Hernán A. Burbano. "Extraction of ultrashort DNA molecules from herbarium specimens." Biotechniques 62, no. 2 (2017): 76-79.

Calderón-Cortés, Nancy, Mauricio Quesada, Horacio Cano-Camacho, and Guadalupe Zavala-Páramo. "A simple and rapid method for DNA isolation from xylophagous insects." International journal of molecular sciences 11, no. 12 (2010): 5056-5064.
